# Assessment of the efficacy of acupuncture and chiropractic on treating Cervical spondylosis radiculopathy: A systematic review and meta-analysis

**DOI:** 10.1097/MD.0000000000017974

**Published:** 2019-11-27

**Authors:** Guang Zuo, Tian-Ci Gao, Bing-He Xue, Chuang-Chuang Gu, Yun-Tao Yan, Yong-Wang Zhang, Rui-Jia Liu, Shuang-Qing Du

**Affiliations:** a Hebei University of Chinese Medicine; b Hebei Province Hospital of Chinese Medicine Shijiazhuang City, Hebei; c The First Department of Neurology, Dongzhimen Hospital, Beijing University of Chinese Medicine, Beijng, China.

**Keywords:** cervical spondylosis radiculopathy, acupuncture, chiropractic, systematic review

## Abstract

**Background:**

Cervical spondylosis radiculopathy (CSR) is often described as neck pain accompanied with radiating pain and neurologic symptoms, such as numbness, muscle weakness, and diminished reflexes, in 1 or both upper extremities. As people's lifestyle changes and the population ages, the incidence of CSR continues to increase. Many clinical trials have proven that acupuncture and chiropractic has a significant effect in the treatment of CSR. In this systematic review, we aim to evaluate the effectiveness and safety of acupuncture and chiropractic for CSR.

**Methods:**

We will search PubMed, Cochrane Library, AMED, EMbase, WorldSciNet; Nature, Science online and China Journal Full-text Database, China Biomedical Literature CD-ROM Database, and related randomized controlled trials included in the ChinaResources Database. The time is limited from the construction of the library to February, 2019. We will use the criteria provided by Cochrane 5.1.0 for quality assessment and risk assessment of the included studies, and use the Revman 5.3 and Stata13.0 software for meta-analysis of the effectiveness, recurrence rate, and symptom scores of CSR.

**Trial registration number:**

CRD42019119941.

## 1 Introduction

Cervical spondylosis radiculopathy (CSR) is often described as neck pain accompanied with radiating pain and neurologic symptoms, such as numbness, muscle weakness, and diminished reflexes, in 1 or both upper extremities.^[1]^ Cervical disc herniation or spondylosis, resulting in nerve compression and in flammation, may be the cause of CSR. In a survey conducted by Wang et al, among the 1009 cases, 651 cases of cervical spondylosis were detected, with a detection rate of 64.52%, among which nerve root cervical spondylosis accounted for 17.97% in China.^[2]^ Currently, there is no consensus regarding the most effective management strategies for CR. This may be because there exist few large randomized controlled studies and because of unclear diagnostics (no subjective or objective physical examination findings comparable with magnetic resonance imaging verifying the neck origin of radiculopathy).^[3–5]^ Further randomized controlled trials are needed to determine the most efficacious interventions for this population.

Treatment of cervical radiculopathy is often managed through conservative therapies, which includes oral analgesics, oral steroids, cervical traction, manual therapy, acupuncture, and various combinations of these. But treatments are subject to some limitations owing to the quality of evidence and adverse drug reaction. For example, Oral non-steroidal anti-inflammatory drugs are generally used to alleviate severe pain. But, long-term non-steroidal anti-inflammatory drugs use may increase the risk and cause gastrointestinal ulcers, serious cardiovascular events, hypertension, acute renal failure, and worsening of pre-existing heart failure.^[6]^

In the world, many patients with cervical radiculopathy are increasingly turning to specific conservative treatments, including cervical spine manipulation, to relieve their symptoms and reduce the side-effects of medications.^[7–9]^ As one of the complementary and alternative therapies, cervical spine manipulation has been used for several years in China. According to the definition provided in the literatures, cervical manipulation is described as the use of hands applied to the patients, thereafter a rapid high-velocity, low-amplitude thrust directed at the cervical joints, often accompanied by an audible crack.^[10,11]^ The action effects of cervical manipulative therapy have been found or validated in some experiments, such as separation of the facet joints, relaxation of paraspinal muscles, increasing of blood flow and so on.^[12]^ Acupuncture in the treatment of cervical spondylosis select the disease has a local positive reaction “Liuhe point”, in order to dredge the local Qi and blood, with the disease through the same side of the collateral points and the opposite side of the collateral points in the external and internal channels can adjust the imbalance between the internal and external channels, restore the internal and external 2 channels of Qi and blood circle movement of the normal state.^[13]^

However, there is relatively little evidence into the effectiveness and safety of cervical spine manipulation and acupuncture for specific neck pain, including cervical radiculopathy. The following questions were inconclusive:

(1) the effectiveness of acupuncture and cervical manipulation and acupuncture for neck pain with radiculopathy;
(2) the adverse effect of cervical manipulation and acupuncture.^[14–19]^

Therefore, the purpose of this meta-analysis is to evaluate the literature regarding the effectiveness and safety of using cervical spine manipulation and acupuncture in the treatment of cervical radiculopathy.

## 2 Methods

This is a systematic review and ethical approval was not necessary.

### 2.1 Study registration

This systematic review protocol has been registered on PROSPERO as CRD42019119941 (https://www.crd.york.ac.uk/prospero/display_record.php?ID=CRD42019119941).

### 2.2 Eligibility criteria

#### 2.2.1 Type of study

Take acupuncture and chiropractic as main treatment, including RCTs of the control group (effective methods other than acupuncture and chiropractic). Language is limited in Chinese and English. Non-RCTs, quasi-RCTs, case series, case reports, and crossover studies will be excluded.

#### 2.2.2 Types of interventions

*Experimental interventions:* The acupuncture and chiropractic is used as experimental interventions. Other TCM treatments such as the dose-specific Chinese medicine preparation or the combined western medicine will be limited.

*Control interventions:* As for the control interventions, those who accepted simple western medicine can be used as a control intervention or those who did not get any treatment as a blank control would be adopted. However, once they had accepted the therapy of TCM, the trials will be rejected.

#### 2.2.3 Outcomes

The primary outcome measurement will be Visual analog scale (VAS).Visual analogue scale (VAS) the basic method of VAS is to use a wandering scale with a non-scale 10-cm slide on the front, a sliding scale between the “0” end and the “10” end, a “0” score for painlessness, a “10” score for the most unbearable pain, and a “0-10” scale on the back. In clinical use, the side with scale is turned away from the patient, and the patient slides the scale to the corresponding position according to the intensity of pain. There is a specific scale on the back of the pain measuring scale, and the pain degree index can be read directly according to the position of the scale. The clinical evaluation was “0 ∼ 2” as “excellent”, “3 ∼ 5” as “good”, “6 ∼ 8” as “may”, and greater than “8” as “poor”. VAS is simple, effective, relatively objective and sensitive, and is less affected by other factors when expressing pain intensity

#### 2.2.4 Data sources

*Electronic searches:* The electronic search database includes PubMed, Cochrane Library, AMED, EMbase, WorldSciNet, Nature, Science online, and China Journal Full-text Database, China Biomedical Studies CD-ROM Database (CBM), and China Resources Database. The clinical research studies on the therapeutic effect of acupuncture and chiropractic on cervical spondylosis radiculopathy published in domestic and foreign biomedical journals from the establishment of the library to February, 2019 was searched. Based on the standards of the Cochrane Collaboration Workbook of the International Evidence-Based Medicine Center, a manual and computer-based method is used to conduct related studies. The search terms include: Chinese medicine, traditional Chinese medicine, acupuncture and chiropractic, cervical spondylosis radiculopathy, cervical radiculopathy. According to the characteristics of different databases, comprehensive search using keyword was conducted. All search results are determined after multiple searches. We will follow the references included in the studies to incorporate relevant studies as much as possible to avoid missed detection. The search term in the Chinese database is the translation of the above word. The complete PubMed search strategy is summarized in Table 1.

**Table 1:**
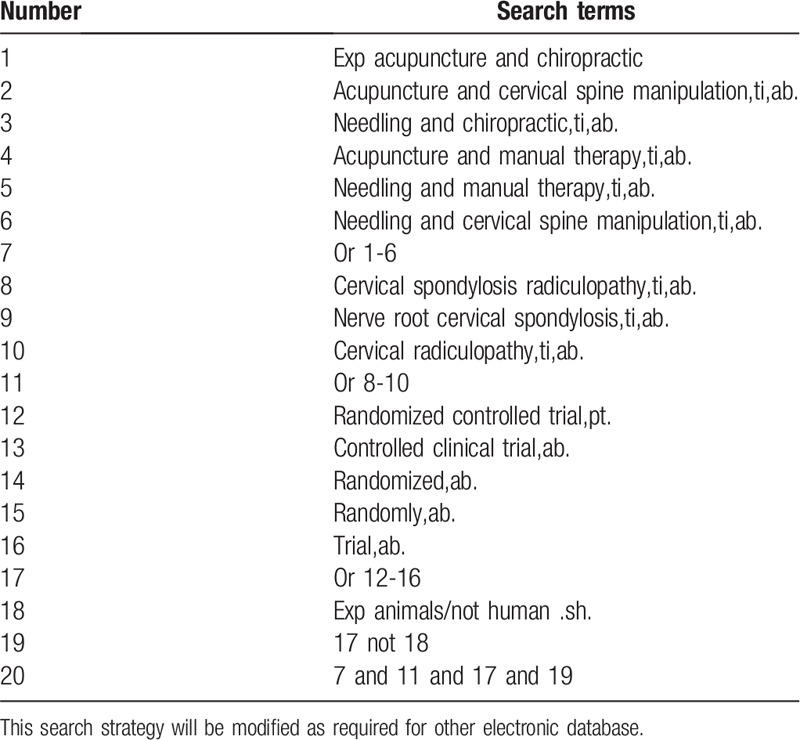
Search strategy used in PubMed database.

*Searching other resources:* The manual search mainly is used for searching relevant studies such as “The Journal of Traditional Chinese Orthopedics and Traumatology” “Chinese Acupuncture and Moxibustion” before the database creation.

#### 2.2.5 Data collection and analysis

**Figure 1:**
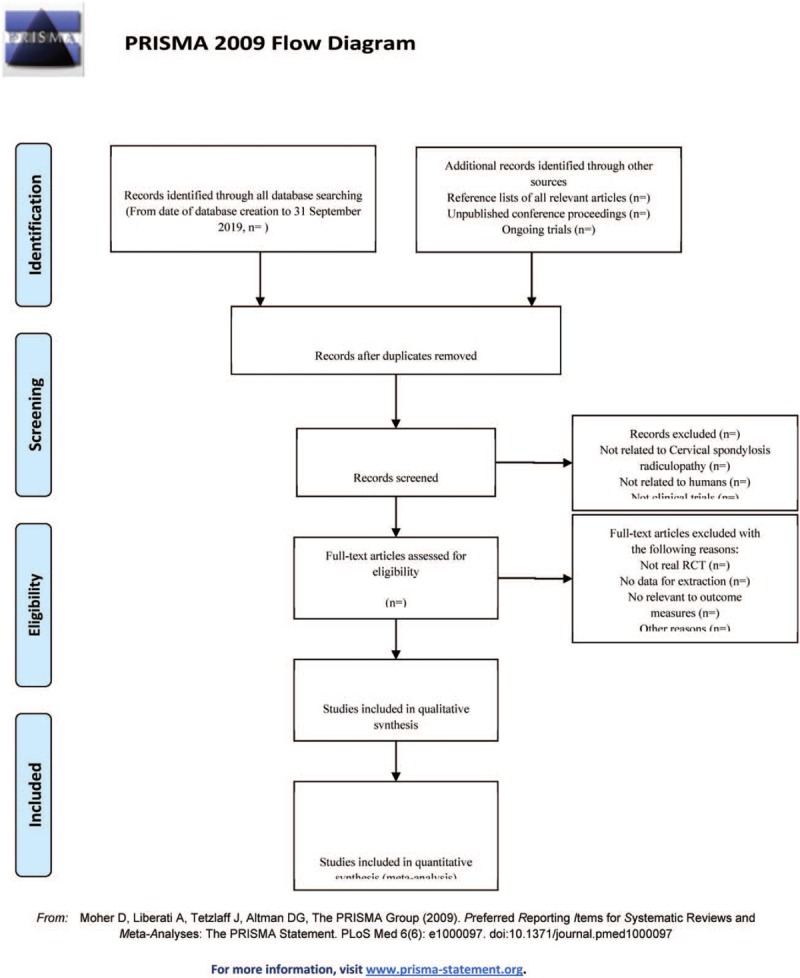
The PRISMA flow chart.

The data collection and analysis was conducted as follows: applying the EndnoteX7 software to manage the included references. Two qualified evaluators independently screened the titles and abstracts of the selected studies, excluding duplicates and documents that did not significantly conform to the study. The second screening of the studies: screening out unqualified studies such as case report, theoretical discussion, and nonconformance of interventions was done. After preliminary evaluation, the remaining studies to further screen out the unqualified studies such as ordinary control study, no control group, no random grouping, no outcome index, and data mine equivalent were carefully read. For the studies that could not be determined whether to be included or not, it was decided by 2 researchers. If the opinions were not uniform, third-party expert was asked. A clinical RCT was finally included in the study. Information and data extraction for the final included documents: the extracted data and information included the test methods of the study, the basic information of the included cases, the observation period, the intervention methods of the treatment group and the control group, the observation indicators, and the test results (Fig. 1).

#### 2.2.6 Risk of bias

The studies quality assessment applies the bias risk assessment tool recommended by Cochrane to evaluate the quality of all included studies and the risk of bias. The assessment will include sequence generation, allocation concealment, blinding of participants, personnel and outcome assessors, incomplete outcome data, selective outcome reporting, and other sources of bias. The risk of high and low bias is expressed as “high risk” and “low risk,” respectively. The information provided in the study is inaccurate or does not provide sufficient information for the bias assessment to be expressed as “unclear risk.” The above content evaluation is independently evaluated by 2 researchers. If there are different opinions, the discussion will be conducted. If there are still differences, a third appraiser will be consulted. Otherwise, the Cochrane Professional Group will be consulted.

#### 2.2.7 Statistical analysis

The meta-analysis in this study will use Rev Man5.3 and Stata13.0 statistical software. Heterogeneity tests will be used for the included experimental studies. The numerical variable will be expressed as the normalized mean difference with a confidence interval (CI) of 95%. The heterogeneity of each pair-wise comparison will be tested by chi-square test (test level a = 0.1). If there is no heterogeneity, a fixed-effect model will be used. If there is significant heterogeneity between a set of studies, we will use a random-effects model (REM) for meta-analysis. We will explore the reasons for the existence of heterogeneity from various aspects such as the characteristics of the subjects and the degree of variation of the interventions. The source of heterogeneity is further determined by means of sensitivity analysis.

#### 2.2.8 Publication bias

If a result of a meta-analysis contains more than 10 articles and above, we will use a funnel plot to test the risk of publication bias. Quantitative methods such as Begg testing and Egger testing will be used to help assess publication bias in the application.

*Quality of evidence:* The Grading of Recommendations Assessment, Development and Evaluation (GRADE) method will be used to assess the quality of evidence for key outcomes. This assessment will be conducted through a Guideline Development Tool (GRADEpro GDT, https://gradepro.org/).

## 3 Discussion

In China, the accelerated pace of life and the popularity of electronic products have boosted the incidence of cervical spondylosis year by year. What is also worth mentioning is that the greater the social, economic and psychological pressure, the more serious the illness. Moreover, it may produce other serious negative effects. As a result, more and more cervical spondylosis patients are reluctant to choose surgery as -the therapy because of fear.^[7–9]^ What they prefer is a kind of more peaceful intervention, such as acupuncture and spinal acupressure therapy, to reduce the side effects of drug or surgery and reduce fear. In order to help the patients recover, an effective treatment solution, including conservative treatment and surgical treatment, is required.^[20]^ However, surgery involves risks, so surgical treatment should only be considered when conservative treatment fails.^[21]^ Specific measures of conservative treatment include drugs, immobilization, physical therapy, manipulation, traction and transcutaneous electrical nerve stimulation,^[22]^ but the results are variable because most conservative treatments have not been strictly tested by randomized controlled trials.^[23]^

Up to now, there are some scholars who have done a systematic review and meta-analysis on cervical manipulation in the treatment of cervical spondylotic radiculopathy, and it is concluded that cervical manipulation generates a favorable effect in easing patients’ pain,^[24]^ among which acupuncture therapy delivers impressive performance in analgesia. In a randomized clinical trial conducted by Cohen, it has been proved that acupuncture has the same analgesic effect as drug therapy and is an acceptable and safe analgesic method. However, it is worth noting that there is no treatment method that can provide perfect acute analgesic effect.^[25]^ In clinical conservative treatment, physicians need an alternative, acceptable and effective treatment with little side effect. In China, In China, some scholars have studied the clinical reports on the use of acupuncture in combination with cervical manipulations in the treatment of cervical spondylotic radiculopathy. The clinical trial conducted by Lin et al. combined acupuncture with cervical manipulation to treat cervical spondylotic radiculopathy and it proved to be an intervention method with favorable curative effect.^[14–19]^ Unfortunately, there is still a gap in the evaluation of efficacy and safety. This study will be the first systematic evaluation of the effectiveness and safety of acupuncture combined with cervical manipulations in the treatment of cervical spondylotic radiculopathy. We will strictly carry out the analysis in accordance with the plan, reduce bias as much as possible, and ensure the accuracy of the evaluation.

With the deepening of understanding of cervical radiculopathy and its complications, the trials and clinical reports of acupuncture and cervical spine manipulation of cervical radiculopathy have gradually increased. Whether it is syndrome differentiation or special disease, acupuncture and cervical spine manipulation has achieved good results in the treatment of cervical radiculopathy. To the best of our knowledge, there has been no comparison of the efficacy and safety of acupuncture and cervical spine manipulation for the treatment of cervical radiculopathy in recent years. Therefore, we will compare the effectiveness and safety of acupuncture and cervical spine manipulation in the treatment of cervical radiculopathy by applying systematic evaluation and meta-analysis. The results of this study can provide a possible ranking for acupuncture and cervical spine manipulation of cervical radiculopathy. We hope that the results will provide clinicians with the best options for treatment.

## Author contributions

**Data curation:** XYG, GZ, TCG

**Formal analysis:** GZ, TCG

**Funding acquisition:** SQD, RJL

**Project administration:** SQD

**Supervision:** CCG, YTY

**Validation:** BHX, YWZ

**Writing – original draft:** GZ, TCG

**Writing – review & editing:** SQD, RJL
